# Evaluation of the accuracy of oscillometric non-invasive blood pressure measurement at the ankle in children during general anesthesia

**DOI:** 10.1007/s10877-023-01025-1

**Published:** 2023-05-11

**Authors:** Reham M Gamal, Maha Mostafa, Ahmed M Hasanin, Suzan Adlan Khedr, Ahmed Salah Abdelgalil, Mamdouh Mahmoud Elshal

**Affiliations:** 1 Department of Anesthesia and Critical Care Medicine, National Cancer Institute, Cairo University, Cairo, Egypt; 2 Department of Anesthesia and Critical Care Medicine, Faculty of Medicine, 01 Elsarayah street, Elmanyal, Cairo 11559 Egypt

**Keywords:** Non-invasive blood pressure, Invasive blood pressure, Hypotension, Ankle, Children, Surgery

## Abstract

This study aimed to evaluate the accuracy of oscillometric blood pressure measurement at the ankle in children using invasive blood pressure as reference standard. This prospective observational study included children undergoing noncardiac surgery. Paired radial invasive and ankle non-invasive blood pressure measurements were obtained. Delta blood pressure was calculated as the difference between two consecutive readings. The primary outcome was the mean bias and agreement between the two methods using the Bland-Altman analysis. The ISO standard was fulfilled if the mean bias between the two methods was ≤ 5 ± 8 mmHg. Other outcomes included the trending ability of ankle blood pressure using the four-quadrant plot and the accuracy of ankle measurement to detect hypotension using area under receiver operating characteristic curve (AUC) analysis. We analyzed 683 paired readings from 86 children. The mean bias between the two methods for systolic, diastolic, and mean blood pressure (SBP, DBP, MAP) was − 7.2 ± 10.7, 4.5 ± 12.8, and − 1.8 ± 8.2 mmHg, respectively. The concordance rate of ankle blood pressure was 72%, 71%, and 77% for delta SBP, DBP and MAP, respectively. The AUC (95% confidence interval) for ankle MAP ability to detect hypotension was 0.91 (0.89–0.93) with negative predictive value of 100% at cut-off value ≤ 70 mmHg, We concluded that in pediatric population undergoing noncardiac surgery, ankle blood pressure was not interchangeable with the corresponding invasive readings with the ankle MAP having the least bias compared to SBP and DBP. An ankle MAP > 70 mmHg can exclude hypotension with negative predictive value of 100%.

## Introduction

Blood pressure measurement is a standard practice during anesthesia; and maintaining adequate blood pressure is important to maintain organ perfusion [1]. The gold standard for blood pressure measurement is through an arterial catheter [2]. However, being invasive, with possible serious complications, intra-arterial blood pressure monitoring is reserved for patients with significant cardiac comorbidity and/or those undergoing major surgery with major fluid shift. Hence, non-invasive blood pressure monitoring is the standard practice during uncomplicated surgery [2] with the arm being the standard site for application of the cuff. However, it is sometimes infeasible to use the arm for measurement due to various reasons such as interference with surgical site or vascular access. In children during surgery, the arm is commonly inaccessible to the anesthetist especially when the operative site is at the head, the neck, or the upper trunk. Therefore, using the ankle for blood pressure measurement in children, if proved accurate, could be a practical alternative to the arm with an additional advantage for being less disturbing to the child when awake [3].

Previous studies evaluated the accuracy of blood pressure at the ankle using arm non-invasive blood pressure measurement as the reference standard [3–5] and reported a large deviation between the two sites of measurement. However, data regarding the accuracy of ankle blood pressure measurement in relation to invasive blood pressure in pediatric population are lacking. In this study, we aimed to evaluate the accuracy of non-invasive ankle blood pressure using real-time invasive blood pressure measurement as the reference standard.

## Patients and methods

This prospective observational study was conducted in a University Hospital after institutional Ethics Committee approval (Date: May 25, 2021, No: 2101-501-006) from June 2021 to June 2022. Written informed consent was obtained from patient’s guardian before the enrolment.

We included children aged 3–14 years, American Society of Anesthesiologist classification of I-III, scheduled for elective non-cardiac surgery under general anesthesia that requires invasive blood pressure monitoring.

Children with any of the following morbidities: congenital heart disease, severe valvular disease, coarctation of the aorta, arrhythmias, peripheral vascular diseases, and deep venous thrombosis were excluded from the study. Children with lower limb edema, scarring, or previous operations or fractures at the ankle were also excluded.

Upon arrival to the operating room, routine monitors were applied (arm non-invasive blood pressure, electrocardiogram, and pulse oximetry). After induction of general anesthesia, a 22-G radial arterial catheter was inserted, and connected through a fluid filled system to a pressure transducer placed and zeroed at the level of the mid-axillary line. After establishing the invasive blood pressure monitor, the arm cuff was disconnected, and another cuff was applied to the ankle. The ankle cuff size was selected according to the recommendations of the American Heart Association for arm cuff (cuff length and width were 80% and 40% of ankle circumference, measured just above the malleoli, respectively) [6]. The cuff sizes (Dräger, Drägerwerk AG & Co. KGaA, Germany) were as follow; infant cuff for ankle circumference range of 8–13 cm, child cuff for ankle circumference range of 13–19 cm, and small adult cuff for ankle circumference 18–25 cm. All invasive and non-invasive blood pressure measurements were obtained by Infinity® C700 (Drägerwerk AG & Co. KGaA, Germany).

An independent researcher was responsible for collecting the data of invasive and non-invasive blood pressure. The invasive blood pressure measurement was recorded as the reading just after the onset of noninvasive blood pressure cuff inflation. Multiple paired measurements were obtained during the course of the surgery.

For evaluation of the trending ability of the ankle blood pressure measurement, the difference between each two successive readings was calculated (excluding those taken at short interval < 1 min).

All paired blood pressure measurements were obtained while maintaining the ankle at the level of the mid-axillary line using a pillow if needed. Anesthetic and hemodynamic management were according to the discretion of the attending anesthetist.

The primary outcome was the mean bias and agreement between invasive- and ankle non-invasive blood pressure measurements (MAP, systolic blood pressure [SBP], and diastolic blood pressure [DBP]).

Secondary outcomes were the number of paired readings with difference of ≤ 5, 10, and 15 mmHg between invasive and non-invasive blood pressure. Demographic data namely age, sex, weight, height, and ankle circumference were collected and the effect of age, sex, and ankle circumference on the magnitude of bias between the two methods was reported. The trending ability of non-invasive blood pressure in relation to invasive blood pressure was also reported.

The accuracy of ankle MAP to detect hypotension (defined as MAP < 5th percentile for the age) was also calculated. Since MAP 5th percentile in our cohort ranged between 52 and 56 mmHg [7], we used a conservative threshold of hypotension in the current study to be MAP < 56 mmHg.

### Statistical analysis

#### Sample size

The Universal Standard for the validation of blood pressure measuring devices by the Association for the Advancement of Medical Instrumentation/European Society of hypertension/International Organization for Standardization (AAMI/ESH/ISO) requires at least 85 subjects for validation of a non-invasive blood pressure device. The number was rounded to 100 subjects to account for the risk of non-invasive blood pressure failure [8].

#### Statistical analysis

Statistical package for social science (SPSS) software, version 26 for Microsoft Windows (SPSS Inc., Chicago, iL, USA), Microsoft Office Excel 365 (Microsoft Corp, Redmond, WA) and MedCalc software V 14 (MedCalc Software bvba, Ostend, Belgium) were used for data analysis. Continuous data were tested for normality using the Shapiro-Wilk test and were presented as mean ± standard deviation or median (quartiles) as appropriate. The Bland-Altman analysis, adjusted for multiple measurements per subject, was conducted to calculate the mean bias and 95% limits of agreement between invasive and non-invasive readings. The ISO standard was fulfilled if the mean bias between the two techniques was ≤ 5 mmHg and its standard deviation was ≤ 8 mmHg [9]. A generalized estimating equation model was constructed to assess the effect of sex, age, and ankle circumference on the magnitude of bias (the absolute difference between invasive and non-invasive blood pressure measurement). Comparison between SBP, DBP, and MAP magnitude of bias was performed using the analysis-of-variance test with post-hoc Tukey test. The trending ability of ankle non-invasive blood pressure measurement in relation to the invasive blood pressure trending values was presented in four-quadrant plot, and the concordance rate was calculated as the ratio of the number of points in the upper right and lower left quadrants in relation to the total number of points in all four quadrants. A central exclusion area was identified using trending values < 5 mmHg. Area under receiver operating characteristic curve (AUC) analysis for the ability of ankle MAP to detect hypotension was performed. The best cut-off value was identified using the Youden index and the corresponding negative and positive predictive values were reported.

- The STROBE guidelines were applied when writing this manuscript.

## Results

**Fig. 1:**
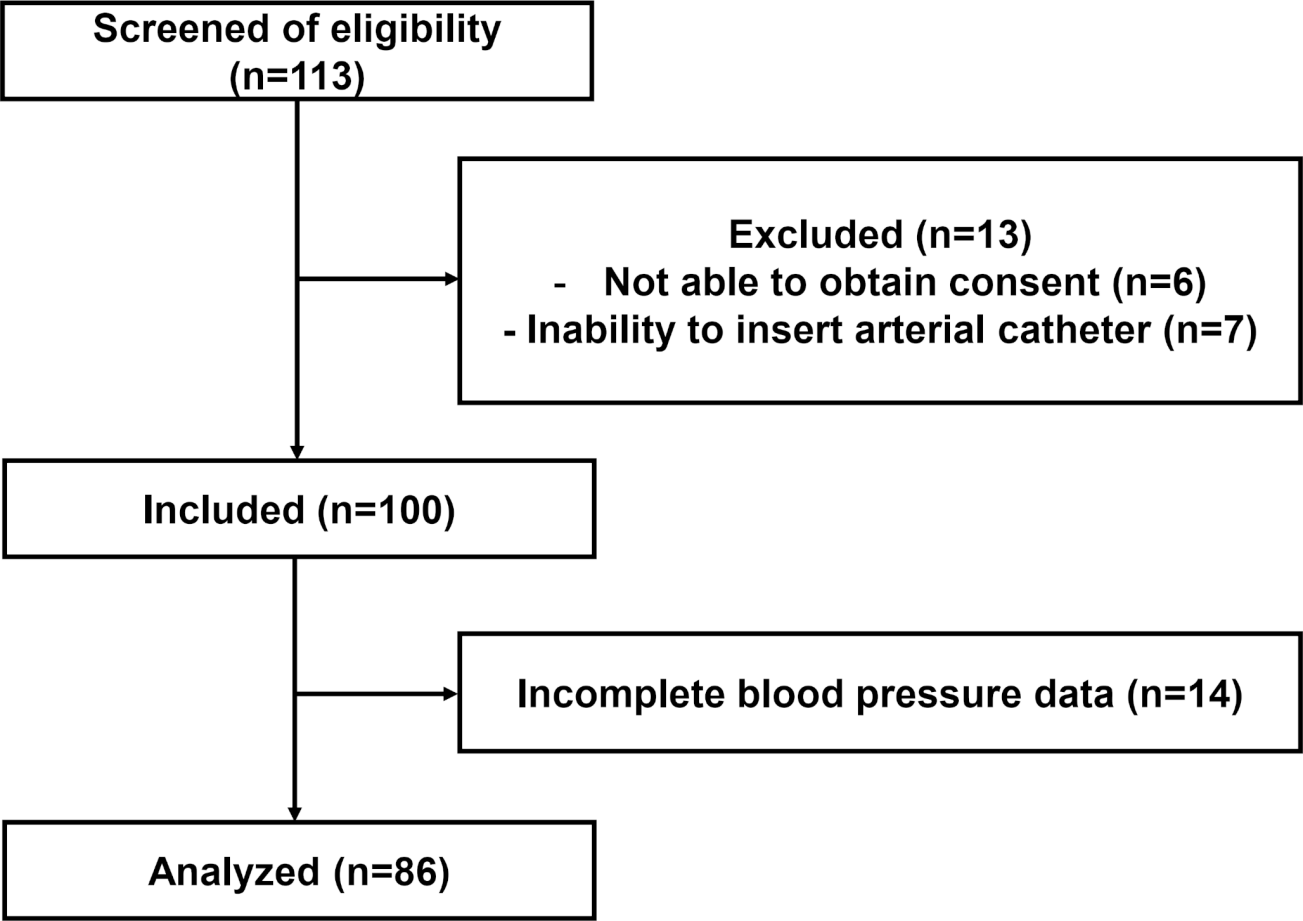
Patients’ enrolment flow chart

One-hundred and thirteen patients were screened for eligibility, 13 patients were excluded (6 patients were excluded due to failure to obtain consent, 7 patients were excluded due to failure to insert a radial artery catheter) and 100 patients were included for this study. Fourteen patients were excluded from the analysis due to incomplete data and 86 patients with 683 paired readings were available for the calculation of the bias and agreement between the two measurement sites. (Fig. 1) The number of paired readings used for assessment of the trending ability was 511 readings.

The median (quartiles) age of the participants was 6 (4, 9) years, and 46/86 (54%) of the participants were males. (Table 1) All the included children were undergoing major head and neck procedures requiring invasive blood pressure monitoring.

**Table 1:** Demographic data. Data presented as mean ± standard deviation, median (quartiles) and frequency (%)

Age (years)	6 (4, 9)
Male sex	46/86 (54%)
Weight (kg)	24 (17, 38)
Height (cm)	123 (107, 140)
Ankle circumference (cm)	15.3 ± 4.5
Mean invasive blood pressure ± standard deviation (min – max)	
SBP	101 ± 13 (54–147)
DBP	64 ± 12 (27–106)
MAP	78 ± 12 (40–123)

DBP: diastolic blood pressure, MAP: mean arterial pressure, SBP: systolic blood pressure

**Fig. 2:**
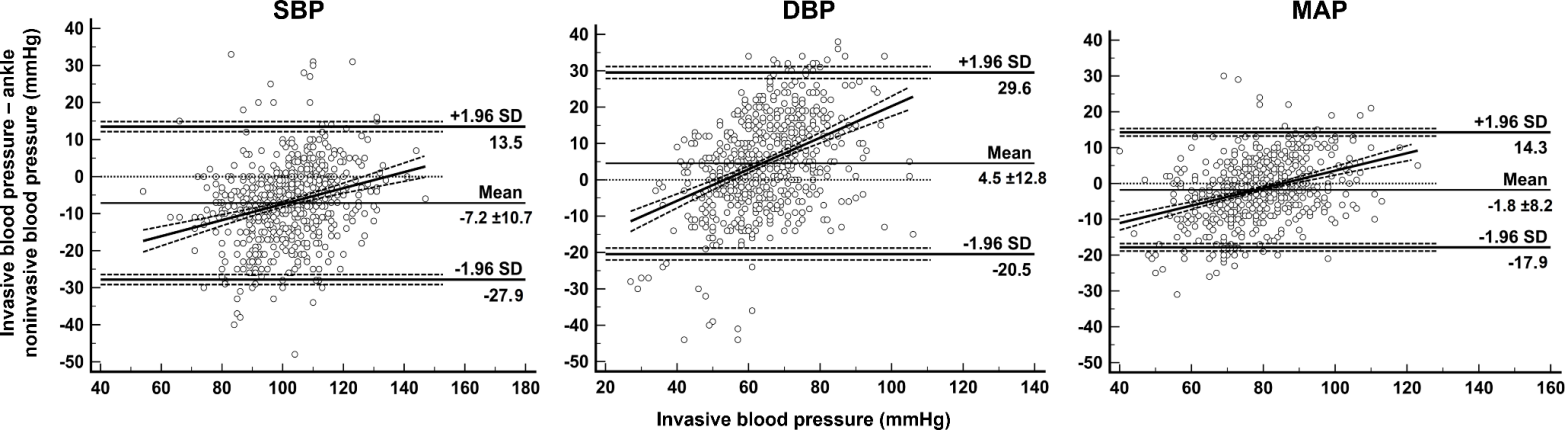
Bland-Altman analysis for all invasive and ankle non-invasive blood pressure measurement (n = 679). Horizontal solid lines represent the mean bias and its 95% limits of agreement. The oblique line represents the regression line linking the bias and the invasive blood pressure; SBP: y =-29.1 + 0.2 x; constant bias of -29.1 mmHg, p < 0.001; proportional bias of 0.2, p < 0.001. DBP: y = -23.2 + 0.4 x; constant bias of -23.2 mmHg, p < 0.001; proportional bias of 0.4, p < 0.001. MAP: y = -20.8 + 0.2 x; constant bias of -20.8 mmHg, p < 0.001; proportional bias of 0.2, p < 0.001. DBP: diastolic blood pressure, MAP: mean arterial pressure, SBP: systolic blood pressure

The mean bias between the two methods was − 7.2 ± 10.7, 4.5 ± 12.8, and − 1.8 ± 8.2 mmHg for SBP, DBP, and MAP respectively. (Fig. 2)

The ankle blood pressure measurement showed both significant systematic and proportional bias in relation to the invasive blood pressure measurement resulting in overestimation of low invasive blood pressure reading and underestimation of high invasive blood pressure reading. (Fig. 2)

The number of paired readings with difference ≤ 10 mmHg was 438 (65%), 380 (56%), and 556 (82%) for SBP, DBP, and MAP, respectively. (Table 2)

**Table 2:** Number of readings with Absolute difference between the two methods of ≤ 5, 10, 15 mmHg

	≤ 5mmHg	≤ 10mmHg	≤ 15mmHg
SBP	241 (35%)	441 (65%)	539 (79%)
DBP	250 (37%)	384 (56%)	521 (76%)
MAP	356 (52%)	559 (82%)	632 (93%)

DBP: diastolic blood pressure, MAP: mean arterial pressure, SBP: systolic blood pressure

The magnitude of bias between the two methods was the lowest for MAP (6.5 ± 5.6 mmHg) in comparison to the SBP (9.9 ± 8.0 mmHg) and DBP (10.6 ± 8.5 mmHg), P-value < 0.001 for both comparisons. The generalized estimating equation analysis on the effect of patient’s characteristics on the magnitude of bias between the two methods revealed that only SBP bias is affected by the child age. (Table 3)

**Table 3:** Effect of patient’s characteristics on the magnitude of mean bias between the two methods

	SBP bias	DBP bias	MAP bias
	Beta estimate (95% confidence interval)		
Male sex P-value	2.73 (-0.14 to 5.60) 0.062	0.02 (-3.25 to 3.28) 0.992	1.56 (-0.54 to 3.66) 0.146
Age	0.64 (0.21 to 1.07)	0.20 (-0.32 to 0.72)	0.10 (-0.25 to 0.454)
P-value	0.004	0.445	0.573
Ankle circumference	-0.01 (-0.05 to 0.04)	0.03 (-0.04 to 0.10)	0.02 (-0.04 to 0.82)
P-value	0.716	0.716	0.447

DBP: diastolic blood pressure, MAP: mean arterial pressure, SBP: systolic blood pressure

**Fig. 3:**
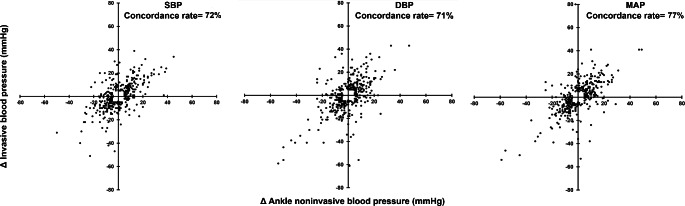
Four-quadrant scatter plot for the trending values of the ankle non-invasive blood pressure in relation to invasive blood pressure. DBP: diastolic blood pressure, MAP: mean arterial pressure, SBP: systolic blood pressure

The four-quadrant plot analysis for the trending ability of the ankle non-invasive blood pressure, revealed a concordance rate of 72%, 71%, and 77% for delta SBP, delta DBP, and delta MAP, respectively. (Fig. 3)

The number of hypotensive readings (MAP < 56 mmHg) was 16 (2%). The AUC (95% confidence interval [CI]) for the ability of ankle MAP to detect hypotension was 0.91 (0.89–0.93), with sensitivity (95% CI) of 88% (62–98%), specificity (95% CI) of 82% (79–85%), positive predictive value (95% CI) of 11% (6–17%) and negative predictive value (95% CI) of 100% (99–100%) at ankle MAP of ≤ 70 mmHg,

## Discussion

In this report, we evaluated ankle non-invasive blood pressure using invasive blood pressure measured at the radial artery as the reference standard in children undergoing noncardiac surgery. We found that ankle measurement did not satisfy the ISO standard [9] as the mean bias ± standard deviation was > 5 ± 8 mmHg with poor trending ability for all outcomes, namely SBP, DBP and MAP. However, the ankle non-invasive measurement seems the most convenient if the MAP was used as it had the lowest bias and was the closest to satisfy the ISO standard. The mean bias in ankle MAP was − 1.8 mmHg which is accepted in the ISO standard and the standard deviation was 8.2 mmHg which is slightly above the standard. Furthermore, the number of paired MAP readings with difference > 10 mmHg was 18%; a value which fulfils the requirements of the European society of hypertension [10]. The excellent accuracy of ankle MAP in excluding hypotension (negative predictive value of 100%) also favors the use of MAP whenever the cuff was applied at the ankle.

The low accuracy of both SBP and DBP at the ankle is explained by the pulse pressure amplification phenomenon, which is the progressive increase of pulse pressure from central to peripheral arteries, resulting in increased SBP and, to a lesser extent, decreased DBP, while the MAP is the least affected [11].

We evaluated the effect of patient’s characteristics, namely the age, male sex, and ankle circumference, on the magnitude of bias between ankle and invasive measurement and found that the difference between the two methods increased with the child age for SBP only. This could contribute to the inaccuracy of ankle SBP in this cohort. On the other hand, both DBP and MAP were not affected by patient’s characteristics.

Previous reports in pediatric population did not support the accuracy of ankle blood pressure [3, 4]; however, these studies [3, 4] used arm non-invasive blood pressure as the reference standard. There is an acceptable bias between the arm noninvasive blood pressure measurement and the invasive measurement [12]; however, this bias becomes significant when the arm is used as the reference standard instead to validate the ankle blood pressure.

Hayes et al. [13] investigated the accuracy of ankle blood pressure in relation to invasive blood pressure as the reference standard in patients undergoing cardiothoracic surgery and reported that ankle blood pressure significantly deviated from the invasive measurement. The authors reported a higher bias and wider limits of agreement than ours specially for the MAP (mean bias [95% limits of agreement]: -5 ± 11 [-26 to 16] mmHg), and the number of MAP readings with > 10 mmHg bias was 27%. However, several differences between our study and Hayes et al’s that might explain the differences between the findings of both [13]: the type of patients (we included patients without cardiac anomalies), type of surgery (noncardiac surgery), the larger sample size (86 versus 30 children) and subsequently the larger number of analyzed readings in our study. Finally we evaluated the accuracy of ankle blood pressure to detect hypotension using the AUC analysis.

We also report that the ankle measurement underestimated high invasive readings and overestimated low invasive readings. This finding is supported by previous studies that evaluated oscillometric devices [14]. Therefore, it is proposed that the threshold for hypotension should be higher than the usual when an oscillometric device is in use [15]. In keep with this statement, we reported that the best cut-off value for ankle MAP to detect hypotension (defined as invasive MAP < 56 mmHg) was ≤ 70 mmHg.

Intra-arterial measurement is the gold standard for blood pressure measurement; however, using this invasive and expensive technique is usually reserved for patients undergoing major surgery and/or unstable critical patients [16]. Thus, non-invasive blood pressure monitoring is the commonest method for measurement with the oscillometric-based devices being the principal everyday tools. The arm is the standard site for non-invasive blood pressure measurement; however, sometimes it is not possible to apply the cuff at the arm for being the site of surgery, presence of intravenous access or arteriovenous shunt, or just for being inaccessible due to surgical position. Furthermore, the ankle is more accessible to the anesthetist in case of upper body procedure. The ankle blood pressure is a suggested alternative and has the added advantage of being less disruptive to the child when awake [3]; hence, it can be used in ward, intensive care unit in addition to the operating room. The results of our study suggest that ankle blood pressure is not interchangeable with the invasive measurement in both absolute and trending values. However, ankle MAP measurement was the closest to the invasive measurement and showed good ability in excluding hypotension. The use of the ankle for blood pressure measurement is sometimes the only choice during surgery, when other alternatives are not feasible. According to our findings, we suggest that the MAP is the most appropriate target during patient management, whenever the ankle non-invasive blood pressure was used. The high negative predictive value for hypotension provides useful implications; no intervention would be needed to correct MAP as long it is > 70 mmHg; however, ankle MAP ≤ 70 mmHg dose not confirm hypotension and should be carefully interpreted by the physician.

Our study had some limitations. 1- It was performed in a single center. 2- A single model of blood pressure monitor and non-invasive cuff were used in this study; hence, our findings are limited to this model. 3- We excluded patients with significant cardiac anomalies and arrythmias. More studies needed to evaluate the ankle blood pressure in these populations. 4- We excluded patients less than three years; however, the study’s age range is based on the AAMI/ESH/ISO recommendation of age range when validating noninvasive device in pediatric population [8]. Future studies are needed to evaluate the accuracy of ankle blood pressure in children less than three years. 5- The number of hypotensive readings was low; therefore, future studies are needed to confirm our findings.

## Conclusion

In pediatric population undergoing noncardiac surgery, ankle blood pressure was not interchangeable with the corresponding invasive readings with the ankle MAP having the least bias compared to SBP and DBP. An ankle MAP > 70 mmHg can exclude hypotension with negative predictive value of 100%.

## Data Availability

The data that support the findings of this study are available from the authors upon reasonable request after permission of Cairo University.
